# Anti-MDA5 autoantibodies predict clinical dynamics of dermatomyositis following SARS-CoV-2 mRNA vaccination: a retrospective statistical analysis of case reports

**DOI:** 10.1007/s00296-024-05683-5

**Published:** 2024-08-27

**Authors:** Christian R. Klein, Annkristin Heine, Peter Brossart, Pantelis Karakostas, Valentin Sebastian Schäfer

**Affiliations:** Clinic of Internal Medicine III, Oncology, Hematology, Immune-Oncology and Rheumatology, University Hospital, Bonn, Bonn, Germany

**Keywords:** Dermatomyositis, mRNA vaccination, MDA-5, Autoimmunity, COVID-19, Case report

## Abstract

Since the introduction of mRNA vaccines against SARS-CoV-2, the induction of autoimmunity by mRNA vaccination has been discussed. Several cases of dermatomyositis (DM) associated with mRNA vaccination against SARS-CoV-2 infection have been reported. The question is whether there is a common pathomechanism for the induction of DM by this mRNA vaccination. The aim of this review is to analyse the sample of previously published case reports of DM following COVID-19 mRNA vaccination for common indicators of a possible immune pathomechanism.

In this review, we summarised case reports of DM following mRNA vaccination against COVID-19. We considered this case report landscape as a cumulative sample (*n* = 32) and identified common clinical and molecular parameters in the intersection of case reports and statistically analysed the effect of these parameters on the development of DM.

MDA-5 antibodies seem to play a role in the autoantibody signature after mRNA vaccination. MDA-5-positive DM is statistically more strongly associated with mRNA vaccination and interstitial lung disease/rapidly progressive interstitial lung disease (ILD/RP-ILD) than MDA-5-negative DM. MDA-5-positive DM seems not to be associated with an increased risk of malignancy, whereas MDA-5-negative DM is more strongly associated with malignancy.

Our findings emphasize the potential role of innate antiviral signalling pathways in connecting DM to mRNA vaccination. MDA-5 autoantibodies appear to be predictive of a severe DM progression following mRNA vaccination. There seems to be an association between MDA-5 autoantibodies and paraneoplastic DM post-vaccination. Further studies are required to uncover the underlying mechanisms of autoimmunity triggered by mRNA vaccination.

## Significance and innovations (Key points)

1. DM with positive MDA-5 seems to correlate with mRNA vaccination, whereas DM without MDA-5 doesn’t. This aligns with observed autoimmune responses post-mRNA vaccination.

2. DM without MDA-5 tends to be more paraneoplastic than that induced by mRNA vaccination.

3. MDA-5 autoantibodies could serve as potential indicators to predict severe DM progression following mRNA vaccination.

## Introduction

Dermatomyositis is a systemic autoimmune disease that manifests in the skin and muscles and can be divided into 6 subgroups according to the autoantibody profile (anti-TIF1-γ, anti-NXP2, anti-MDA5, anti-SAE (SUMO-1 activating enzyme), anti-Mi-2-associated and autoantibody-negative DM) with different prognoses [1]. Of particular importance is anti-MDA5-associated DM, which is more commonly associated with interstitial lung disease/rapidly progressive interstitial lung disease (ILD/RP-ILD) and high mortality [2]. MDA5 (melanoma differentiation-associated protein 5) recognises long dsRNA in the cytosol and activates MVAS (mitochondrial antiviral-signalling protein), which can activate signalling pathways with NFκB (nuclear factor ‘kappa-light-chain-enhancer’ of activated B-cells) and IRF3 (Interferon regulatory factor 3) via TRIF (TIR-domain-containing adapter-inducing interferon-β). As part of the inflammasome, MDA5 induces a type 1 interferon response and, alongside LGP2 (Probable ATP-dependent RNA helicase DHX58), is the main sensor of SARS-CoV-2 infection [3–5]. Previous work suggested that COVID-19 mRNA vaccines are potential stimulators of a type 1 interferon immune response and subsequent autoantibody production against MDA5 [6]. The clinical relevance of anti-MDA5 in vaccine-associated DM has been documented in two case series [7, 8]. Despite many case reports of mRNA vaccination-associated DM, there is no consistent clinical picture of this association and possible pathomechanisms to date. In this study, we summarise the case report landscape of DM after mRNA vaccination against COVID-19 as a cumulative sample (*n* = 32) and analyse clinical and molecular parameters to obtain a more accurate picture of DM after mRNA vaccination.

## Materials and methods

### Design and data source

**Fig. 1:**
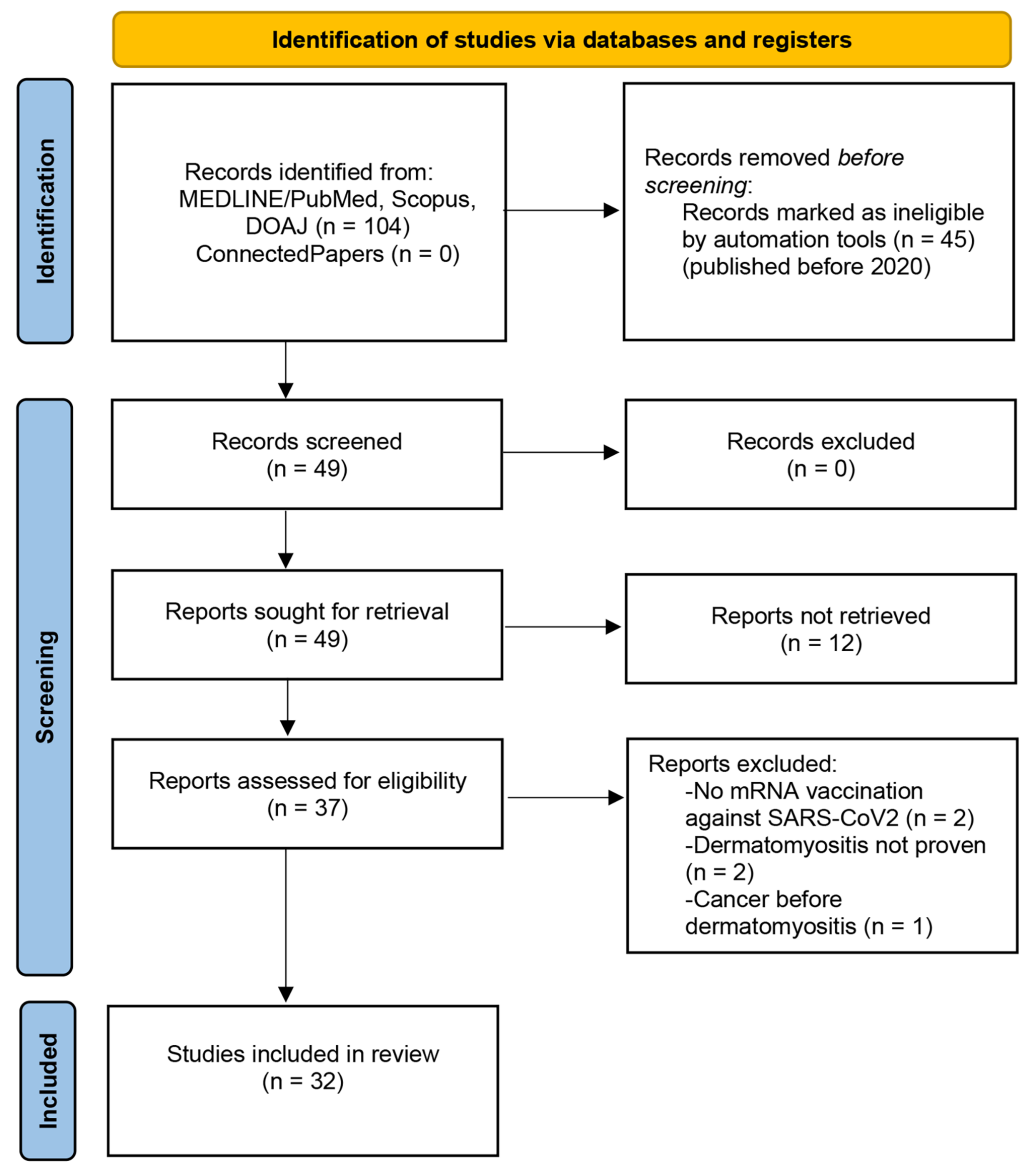
PRISMA flow diagram (Status of the literature search: 07/2024): dermatomyositis following SARS-CoV-2 mRNA vaccination

Initially, a literature search was conducted according to the Preferred Reporting Items for Systematic Reviews and Meta-Analyses (PRISMA) guidelines [9]. As shown in the PRISMA flow diagram (Fig. 1), a literature search using the key words “SARS-CoV2” AND “dermatomyositis” AND “vaccination” and a subsequent hand search of related articles (https://www.connectedpapers.com/) identified 49 case reports, of which 32 were included in the case report analysis [7, 8, 10–27]. As some case series were included, the number of publications is smaller than the number of case reports. Exclusion criteria were non-mRNA vaccination or an unspecified vaccine type against SARS-CoV2, DM after SARS-CoV2 infection, no evidence of DM, a priori evidence of paraneoplastic DM (tumour-associated occurrence *preceding* mRNA vaccination) or evidence of autoantibodies indicative of myositis other than DM. The identified case reports were pooled as one sample and common clinical parameters of the case reports were identified.

### Parameter selection

From the case report landscape, we identified common clinical and molecular parameters for analysis. In addition to the basic parameters of age (years), sex (male/female), mRNA vaccine type (mRNA-1273, BNT162b2), number of vaccinations before the onset of symptoms, latency (days) between the last mRNA vaccination and the first appearance of DM-specific symptoms, number of pre-existing diseases, we extracted the clinical parameters heliotropic erythema, mechanic’s hand, shawl sign, Gottron’s papules, periungual erythema, weight loss, fever, fatigue, dysphagia, dyspnoea, muscle pain, lack of strength/weakness, ILD/RP-ILD from the case reports. Other clinical symptoms of DM were not reported with sufficient frequency or consistency. Common laboratory parameters recorded were creatine kinase (CK [U/L]), aspartate transaminase (AST [U/L]), alanine transaminase (ALT [U/L]) and C-reactive protein (CRP [mg/dL]). In addition, the following autoantibodies were documented in the case report landscape ANA, MDA-5, TIF-1γ, SSA(Ro), Mi-2, ENA, NXP2, RF, SAE-1, CCP, jDM, RNP, SRP, PM/Scl75, PM/Scl100, PL-7, PL-12. Other autoantibodies were not detected. ENA sums the presence of autoantibodies SSA (Ro), SSB (La), RNP, Sm, Scl-70, Jo-1. In addition, the substances used for the treatment of DM, a cancer screening carried out and its result (if applicable, the detection of a malignant disease) were recorded.

### Statistical analysis

Data collected from the case reports were collated into a common matrix. Descriptive analysis was performed using Microsoft Excel 2019 (Microsoft, Redmond, WA, USA), and inductive statistics were performed using Python 5.4.3. Statistical analysis was performed according to the scale level of each parameter. Quantitative data are presented as median and interquartile range (IQR: 25th and 75th percentiles). Qualitative variables are presented as absolute frequencies and relative frequencies (%). The Pearson chi-squared test was used to assess the relationship between two qualitative variables. For smaller samples, Fisher’s exact test was used for categorical variables. Pearson’s phi coefficient φ and Cramer’s V (contingency coefficient) were calculated as measures of the degree of correlation between nominally scaled data. The Shapiro-Wilk test (test statistic W) was used to test for normal distribution (with small sample *n* < 50). Kendall’s τ_K_ and Spearman’s rank correlation coefficient r_s_ were used to examine the correlation between ordinally and nominally scaled data. A Mann-Whitney U test was used to test ordinally and nominally scaled data. Cohen’s d (equal sample size) and Hedges’ g (unequal sample size) were used to quantify effect size. All results are considered significant when *p* < 0.05. All results have been rounded to 3 decimal places.

### Ethical aspects

This case report analysis is a completely retrospective research project, as all the data to be used were already available as journal-published case reports at the time the research question was posed. The data set of the research project does not contain any newly collected clinical data, but is an analysis of already internationally published case reports. According to Section 15 (1) of the Professional Code of Conduct of the North Rhine Medical Association, retrospective epidemiological research projects are exempt from the obligation to consult a medical ethics committee. The Ethics Committee of the Medical Faculty of Bonn and the Ethics Committee of the North Rhine Medical Association agree with this regulation, so that no ethical approval was obtained for this retrospective study in accordance with their guidelines. The work reported in this manuscript was conducted entirely in accordance with EU Regulation 2016/679 (General Data Protection Regulation) and the Declaration of Helsinki.

## Results

### General characteristics of the case report landscape

**Fig. 2:**
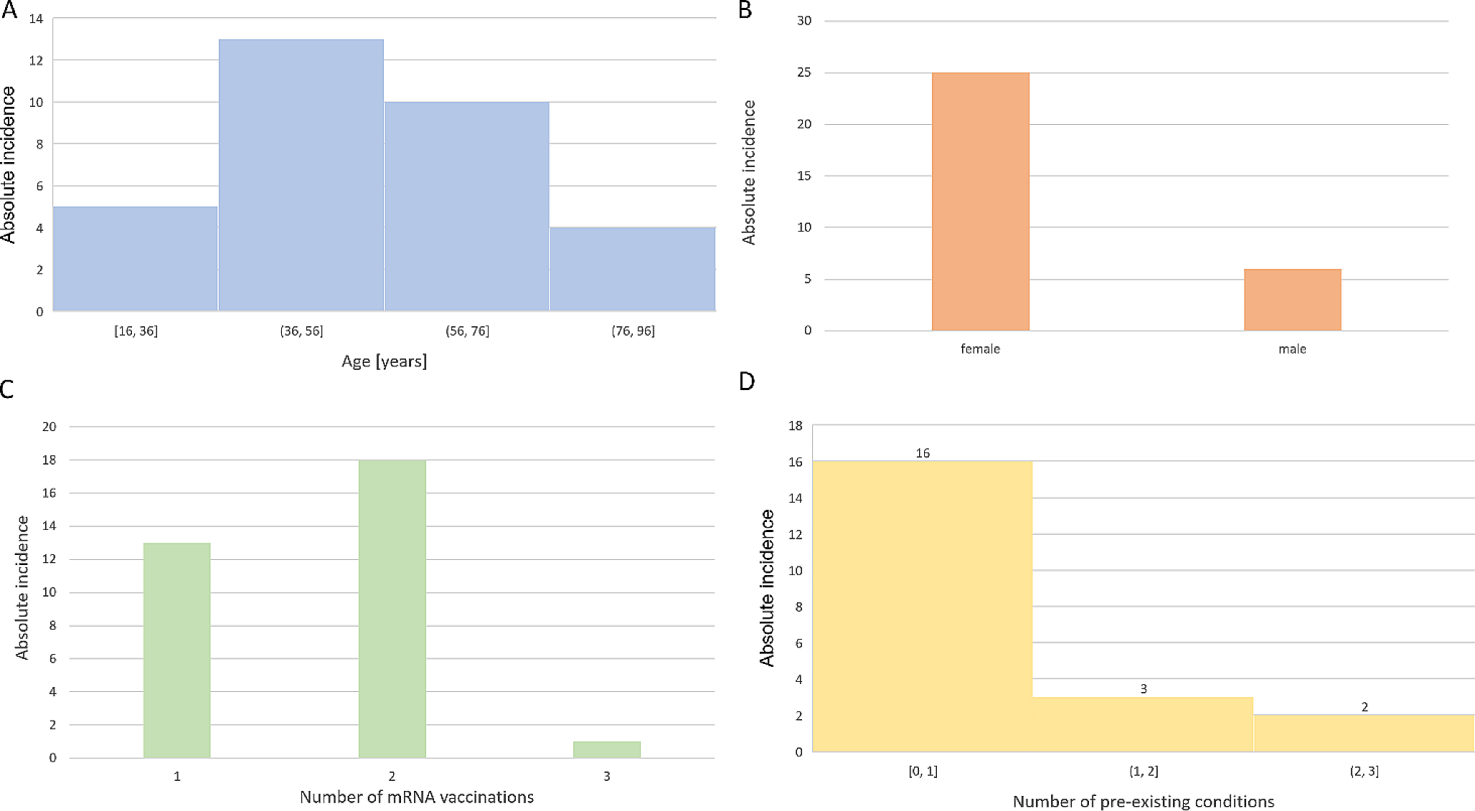
General characteristics of the case report landscape. **A**: Age distribution of the discussed case reports on dermatomyositis following mRNA vaccination. **B**: Absolute incidence of case reports of on dermatomyositis following mRNA vaccination by sex. **C**: Number of mRNA vaccinations before symptom onset of dermatomyositis. **D**: Number of pre-existing conditions in patients in the case report sample. If pre-existing conditions were not reported in the case reports, these patients were not included in the presentation

The general results of the case report landscape are visualised in Fig. 2. The age distribution of the sample follows a normal distribution (W = 0.959, *p* = 0.250) with a mean $\:{\mu\:}_{Age}=\:54.25\:\pm\:\:19.79$ years (Fig. 2A). In the 32 case reports, more than four times as many women as men developed DM (25 women vs. 6 men). In this sense, there is a gynaecotropy, which is known in DM and other autoimmune diseases (Fig. 2B). DM-specific symptoms in the case reports analysed occurred mainly after the 1st and 2nd vaccination; only one case report reported an association between DM and mRNA vaccination after 3 doses of BNT162b2 [22]. Interestingly, the majority of case reports report the onset of DM-specific symptoms after the administration of the second vaccination dose (Fig. 2C). Patients in the case reports analysed predominantly presented with no or very few pre-existing diseases (Fig. 2D). A spurious correlation due to comorbidities as mediator variables therefore seems unlikely.

### Clinical results

**Fig. 3:**
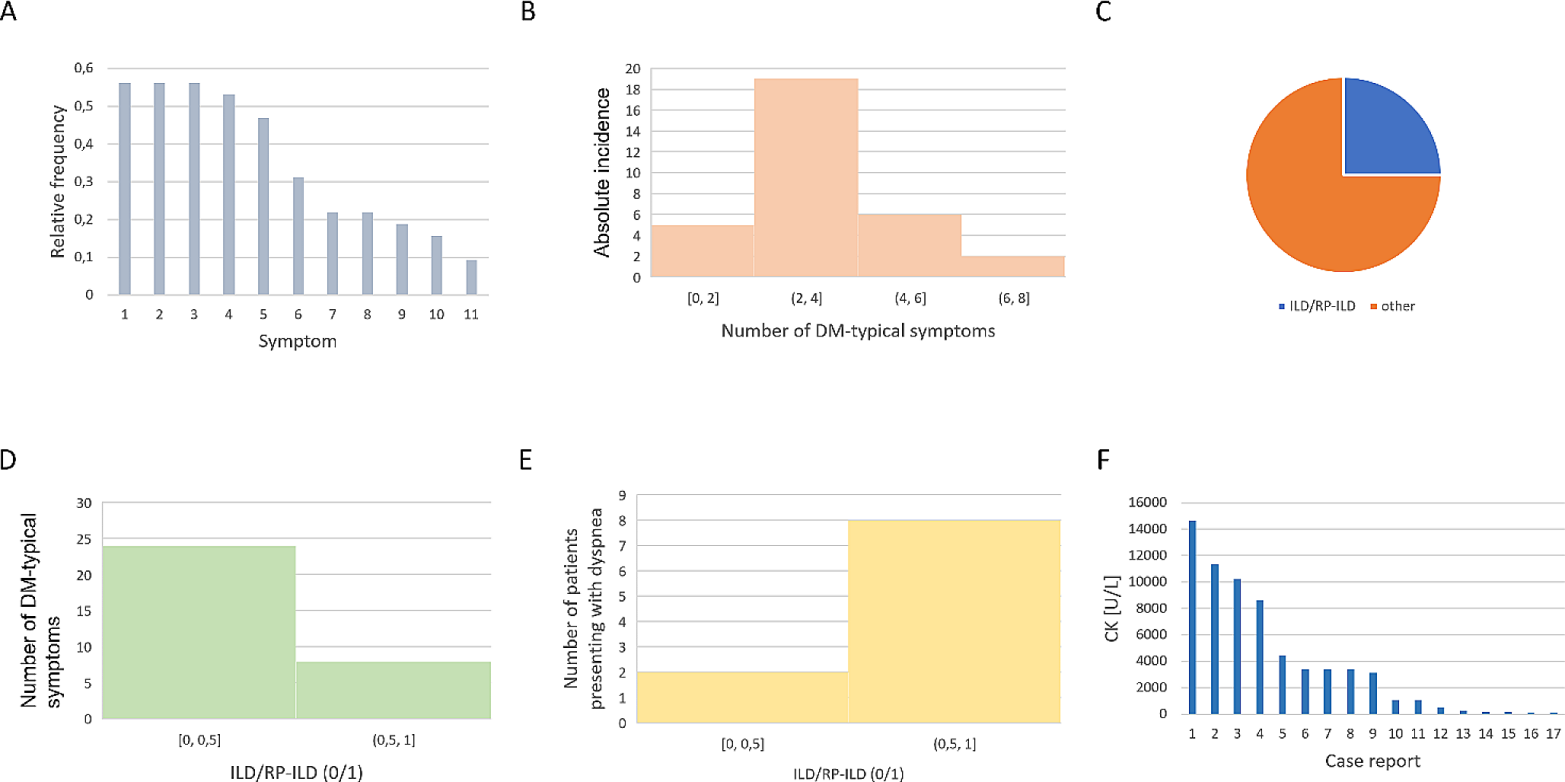
Clinical results. **A**: Relative frequency of DM-typical symptoms. 1: Shawl sign, 2: Gottron papules, 3: Lack of strength/Weakness, 4: Heliotropic erythema, 5: Muscular pain, 6: Periungual erythema, 7: Dysphagia, 8: Dyspnea, 9: Fever, 10: Fatigue, 11: Weight loss. Other DM-typical symptoms were reported less regularly, so they could not be included in our analysis. **B**: Frequency of DM-typical symptoms (Shawl sign, Gottron papules, Lack of strength/Weakness, Heliotropic erythema, Muscular pain, Periungual erythema, Dysphagia, Dyspnea, Fever, Fatigue, Weight loss). **C**: Proportion of patients with interstitial lung disease/rapidly progressive interstitial lung disease (ILD/RP-ILD) on the sample of all case reports. **D**: DM-typical symptoms in patients with and without reported ILD, respectively. Case reports that did not report ILD were assumed not to have been diagnosed with ILD. **E**: Number of patients with dyspnea at initial diagnosis arranged by occurrence of ILD/RP-ILD during progression. **F**: CK levels [U/L] in all case reports (17 of 32 cases) that explicitly documented CK laboratory results. When CK kinetics were reported, the maximum CK value was considered here

The clinical findings of the case report landscape are shown in Fig. 3. The most common DM-specific symptoms in the case reports are shawl sign, Gottron papules, weakness/fatigue, heliotropic erythema, muscle pain, periungual erythema, dysphagia, dyspnoea, fever, fatigue and weight loss. Other DM-typical symptoms were reported less regularly and therefore could not be included in our analysis. Overall, the reported cases of DM after mRNA vaccination were predominantly mild, ambulatory courses; dysphagia and/or dyspnoea were reported in about 20% of cases, and fever or weight loss occurred in less than 20% of cases (Fig. 3A). Most patients initially had few symptoms typical of DM (median: 4, IQR: 2). ILD/RP-ILD was reported in 25% (8 of 32) of cases (Fig. 3C). The diagnosis was made clinically and CT-graphically in each case report. The number of DM-typical symptoms at diagnosis of DM did not predict the occurrence of ILD/RP-ILD (τ_K_ = r_S_= 0.0, p_τK_ = p_rS_ = 1.0; Fig. 3D). The occurrence of ILD/RP-ILD during the course of the disease (if reported in the case reports) predicted the presence of dyspnoea at the initial diagnosis of DM (φ = 0.739, *p* = 2. 016e-06, Fig. 3E); conversely, dyspnoea at first diagnosis of DM is not a significant marker for the development of ILD/RP-ILD in our sample ($\:{\chi\:}^{2}$= 2.4798, *p* = 0.999; V = 0.2239); more data are needed for a more detailed analysis. The serum creatine kinase concentration (CK levels [U/L], serum) of the case report landscape was normal in most cases (18/32, 56.25%) (Fig. 3F); the maximum reported value of 14,659 U/L corresponds to a 73.295-fold increase of the upper limit of normal of 200 U/L [17]. An increase in serum CK concentration in DM is therefore non-specific and not predictive.

### MDA-5 antibodies play a major role in the signature of autoantibodies following mRNA vaccination

**Fig. 4:**
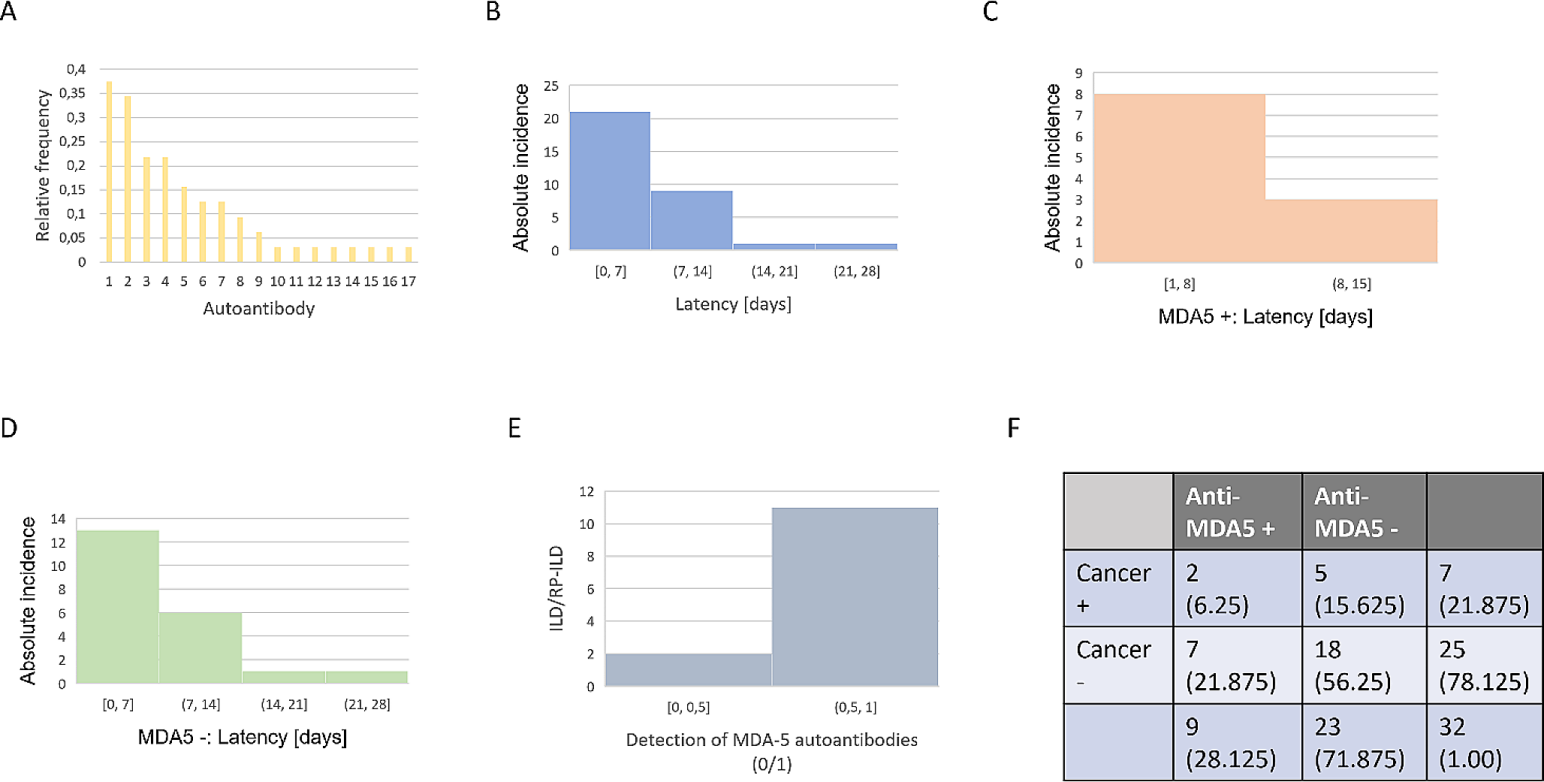
Autoantibody signature after mRNA vaccination and the special role of anti-MDA-5 antibodies. **A**: Relative frequency of Autoantibodies. 1: Antinuclear antibodies (ANA), 2: Anti-Melanoma differentiation-associated protein 5 (MDA-5), 3: Anti-Transcriptional intermediary factor-1γ (TIF-1γ), 4: Sjögren’s-syndrome-related antigen A autoantibodies (SSA(Ro)), 5: Anti-Nucleosome Remodeling Deacetylase complex (Mi-2/ NuRD), 6: Anti-Extractable nuclear antigen (ENA), 7: Anti-Nuclear matrix protein-2 (NXP2), 8: Rheumatoid factor (RF), 9: Anti-Small ubiquitin-like modifier 1-activating enzyme (SAE-1), 10: Cyclic citrullinated peptides (CCP), 11: Anti-Juvenile dermatomyositis antigen (jDM), 12: Anti-Ribonucleoprotein (RNP), 13: Anti-Signal recognition particle (SRP), 14: Anti-Polymyositis/Scleroderma (75 kDa) antigen (PM/Scl75), 15: Anti-Polymyositis/Scleroderma (100 kDa) antigen PM/Scl100, 16: Anti-threonyl-tRNA synthetase (PL-7), 17: Anti-alanyl-tRNA synthetase (PL-12). Other autoantibodies have not been detected. ENA sums the presence of autoantibodies SSA (Ro), Anti-Soluble Substance B-antigen (SSB (La), Anti-Ribonucleoprotein (RNP), Anti- Smith antigen (Sm), Anti- Topoisomerase I (Scl-70), Anti- Histidyl–tRNA synthetase (Jo-1). **B**: Distribution of latency in days between last mRNA vaccination and first appearance of dermatomyositis-specific symptoms. **C**: MDA-5-positive dermatomyositis: Distribution of latency in days between last mRNA vaccination and first appearance of dermatomyositis-specific symptoms. **D**: MDA-5-negative DM: Distribution of latency in days between last mRNA vaccination and first appearance of dermatomyositis-specific symptoms. **E**: Interstitial lung disease/rapidly progressive interstitial lung disease ILD/RP-ILD depending on the presence of MDA-5 autoantibodies. **F**: Four-way table for the detection of anti-MDA-5 autoantibodies (MDA5+/-) and the detection of cancer in the case of successful cancer screening (Cancer+/-). The absolute frequencies of the sample are given, in brackets the relative frequencies based on *n* = 32

The results of autoantibody serology are shown in Fig. 4. Antinuclear antibodies (ANA, Fig. 4A) are the most frequently reported in the case report landscape. This class of antibodies subsumes all autoantibodies against nuclear and some cytoplasmic antigens and is a priori non-specific with respect to autoimmunity and aetiology. Interestingly, the most common ANA detected in the case reports were anti-MDA5 autoantibodies (11/32; 34.375% of case reports). The relatively high frequency of autoantibody detection against TIF-1, NXP2 (Fig. 4A) is suspicious for occult malignancies in paraneoplastic DM and puts into perspective the presumed association of DM with mRNA vaccination in the respective case reports.

#### MDA-5-positive DM is statistically associated with SARS-CoV 2 mRNA vaccination

In the following, we investigated the predictive value of anti-MDA-5 autoantibodies in terms of the temporal association (Fig. 4B, C, D). Irrespective of the autoantibody profile, most DM symptoms occurred within one week, with a maximum of two weeks (Fig. 4B). Beyond that, associations between mRNA vaccination and DM are rarely reported. The latency period (days between the last mRNA vaccination and the first appearance of DM-specific symptoms) of case reports of mRNA vaccination-associated DM mainly occurs in a period of 1–2 weeks after the first or second vaccination.

Our most important result now emerges when differentiating this latency between anti-MDA5-positive and anti-MDA5-negative DM. Comparing these two groups, anti-MDA5-positive DM shows a significantly shorter latency than anti-MDA5-negative DM (Figure C, D; U = 969.5, *p* = 2.409e-10). In this sense, there is a statistically higher association between the latency period between mRNA vaccination and DM in the case of MDA5 positive DM than in the case of MDA5 negative DM. The case reports in this sample were counted as anti-MDA5-positive DM only if anti-MDA5 antibody detection was reported, otherwise as negative. The effect size between the two groups (anti-MDA5 positive/negative) was quantified with Cohen’s d = 0.084 and after adjustment for different group sizes with Hedges’ g = 0.082.

#### Anti-MDA-5 antibodies predict the emergence of ILD/RP-ILD in DM after mRNA vaccination

The detection of anti-MDA5 autoantibodies is also highly predictive of the likelihood of developing ILD/RP-ILD (OR: 12.6, Fisher test, *p* = 0.006). The odds of developing ILD/RP-ILD are 12.6 times higher when anti-MDA5 autoantibodies are detected than when MDA5 is negative (Fig. 4E).

#### MDA-5-positive DM does not increase malignancy risk in the case report landscape

In some of the cases in the case report landscape, cancer screening was performed in DM (despite a suspected pathomechanistic relationship to the previous mRNA vaccination), and in some cases a malignancy was detected (Fig. 4F). However, the presence of anti-MDA5 autoantibodies did not increase the risk of malignancy in our sample (OR: 0.0, Fisher test, *p* = 0.271). If the tumour search was not documented in the case report, it was considered to be negative, also with the rationale that the risk of malignancy was likely to be low a priori for the treating physicians. Thus, MDA5 positivity in DM does not increase the risk of malignancy in the case report landscape. However, with *p* > 0.05, MDA5 positivity does not show a significant risk reduction for malignancy either. A larger sample would be needed to draw stronger conclusions. Conversely, MDA-5 negativity is associated with a slightly increased risk of malignancy, but this effect is not significant at α = 0.05 (OR: 2.833, Fisher test, *p* = 0.379).

## Discussion

Since the introduction of the first mRNA vaccines against SARS-CoV 2, the question of autoimmune side effects or even the induction of autoimmunity by mRNA vaccines has been discussed. So far, autoimmune diseases following mRNA vaccination have only rarely been reported. In this review, we pooled previously published case reports of dermatomyositis following SARS-CoV 2 mRNA vaccination as a cumulative sample to identify common parameters as indicators of a possible pathomechanism.

Independently of the results presented here, the safety of individual SARS-CoV 2 mRNA vaccines with respect to autoimmune diseases (other than DM) has been documented in large samples [28].

### Discussion of the method

To the best of our knowledge, the methodological procedure of cumulating previous case reports into a common sample, and subsequently selecting parameters and statistically analysing these data, is new and not yet established as a research method. The prerequisite for this is a sufficiently large number of individually published case reports. With regard to the question of DM after SARS-CoV 2 mRNA vaccination addressed here, statistically significant statements could be made to a large extent (significance level α ≤ 0.05). In this sense, the sample investigated is sufficiently large for the question addressed here. As the case reports in the case report landscape were worthy of publication partly because of their rarity, it is difficult to obtain a cohort comparable to the sample presented here in the context of a prospective study. The case report landscape is therefore a powerful method and data collection tool for this type of question, which is difficult to improve upon. The age distribution (Fig. 3A) and gynaecotropy (Fig. 3B) [29, 30] found are epidemiologically expected; the age distribution of the sample approximately satisfies a normal distribution; gynaecotropy is a well-characterised feature in DM and other autoimmune diseases. The parameters show that the case reports as a collective are informative about the current state of DM research and that the sample can be representative of the population to a certain extent, despite the singularity of the individual cases.

### Discussion of the general characteristics and clinical findings of the case report landscape

The general characterisation of the case report landscape shows that most cases of DM after SARS-CoV 2 mRNA vaccination occurred after the application of the second vaccination dose. This observation is relevant for the assessment of a possible association between DM and mRNA vaccination in routine clinical practice. Beyond the purely descriptive finding, the question arises whether this observed association is based on a common pathomechanism. If this is the case, the disease dynamics of DM after mRNA vaccination seems to be delayed in some cases, possibly sensitised by the application of a first mRNA vaccination dose, and only becoming clinically symptomatic after the second vaccination dose. An association of DM after a third vaccination dose is rarely reported; in clinical practice, such cases should always be critically assessed for an association of DM and mRNA vaccination, as the temporal dynamics of such a prolonged sensitisation seems to be extremely rare, if at all.

Most patients in the case report landscape had no or very few (0–1) previous diseases (Fig. 3D). The possibility of a spurious correlation between DM symptoms and mRNA vaccination, mediated by previous diseases and corresponding medication as a mediator variable, seems unlikely without formal statistical analysis on a larger sample.

Most patients in the case report landscape had relatively few symptoms and few severe courses of ILD/RP-ILD (Fig. 3A, B). Overall, the reported courses were moderate, and in most cases could be managed entirely on an outpatient basis. The finding that the number of DM-related symptoms at diagnosis did not predict the occurrence of ILD/RP-ILD shows a certain independence of the development of ILD/RP-ILD from the pathophysiology of other DM-related symptoms. This can be interpreted as clinical evidence for a molecularly dissociated pathomechanism of ILD development, possibly involving MDA-5. On the one hand, ILD/RP-ILD is a predictor of dyspnoea at initial diagnosis of DM; conversely, dyspnoea is not a predictor of ILD/RP-ILD in the course of the disease in the case report landscape. On the one hand, this is plausible because dyspnoea can occur as a moderate symptom in DM without consecutive ILD/RP-ILD; on the other hand, in ILD, pulmonary symptoms appear early in the course of the disease. For clinical practice, it is therefore relevant to monitor DM patients with dyspnoea at first diagnosis, especially for ILD/RP-ILD, although dyspnoea cannot predict the development of ILD/RP-ILD. To better assess the predictive value of dyspnoea at first diagnosis of DM, more data are needed for more precise statistical analysis.

### Anti-MDA-5 autoantibodies discriminate DM after SARS-CoV 2 mRNA vaccination

Essentially, in the realm of DM case reports following SARS-CoV 2 mRNA vaccination, two subgroups emerge based on the presence of anti-MDA-5 autoantibodies.

1. Anti-MDA-5 autoantibody positive subgroup: Anti-MDA-5 autoantibodies are the most frequently detected autoantibody after ANA (Fig. 5A). In addition to the finding of MDA-5 autoantibodies as a predictive marker, conversely, the frequency distribution (Fig. 5A) does not indicate another autoantibody that appears to be similarly predictive as a marker for DM after SARS-CoV 2 mRNA vaccination. The group of MDA-5-positive cases showed a shorter latency period between the last vaccine application and the onset of DM-specific symptoms (Fig. 4B + C), a significantly more frequent development of ILD/RP-ILD (Fig. 4E) and no increase in the risk of malignancy (Fig. 4F). Overall, this indicates a type 1 interferon-dependent pathomechanism as described in [6], analogous to the antiviral immune response in the context of SARS-CoV2 infection [31]. The results support the initially formulated hypothesis of a pathomechanistic connection between the development of DM and the SARS-CoV2 mRNA vaccination for the MDA5-positive subgroup. The cellular antiviral immune response via MDA5 RNA sensor in human lung epithelium can then be understood as a molecular correlate of the increased risk of ILD/RP-ILD development (Fig. 4E). More experimental work is needed to elucidate the exact pathomechanism.
2. Anti-MDA-5 autoantibody negative subgroup: The group of MDA-5 negative case reports shows a longer latency period between the last vaccination and the onset of DM-specific symptoms (Fig. 4B + D) and only rarely an ILD/RP-ILD development (Fig. 4E). The risk of malignant tumours is discreetly increased in this subgroup. Overall, this subgroup is much more heterogeneous and no common pathomechanism for the development of DM after SARS-CoV 2 mRNA vaccination can be deduced from the case report landscape for this subgroup. The relatively high detection rate of occult malignancies (5/23, 21.739%, Fig. 4F), despite a suspected association with mRNA vaccination by the authors of the case reports, indicates a high incidence of paraneoplastic genesis of this DM.

In summary, anti-MDA-5 autoantibodies show discriminative potential to differentiate DM following SARS-CoV 2 mRNA vaccination, with MDA-5-positive DM suspected to be pathomechanistically related via an MDA-5-MVAS-mediated type 1 interferon response to mRNA vaccine application. MDA-5-negative DM shows a less clear association to SARS-CoV 2 mRNA vaccination with greater heterogeneity in its pathogenesis, including paraneoplastic DM in occult malignancies. Nevertheless, these results do not identify a molecular pathomechanism; rather, they demonstrate a statistical association between the clinical parameters, which must be validated through prospective experimental studies.

In addition, the selection of anti-MDA5 autoantibodies as a predictive marker for DM following SARS-CoV 2 mRNA vaccination is the result of a special case report sample that is only partially representative of a real clinical cohort. The validation of MDA5 autoantibodies as a marker requires external samples, and other antibodies with the same or better marker qualities may exist that could not be detected with the procedure described here because they were not sufficiently reported as a common parameter in the case reports.

### Discussion of related works

Autoimmune side effects of mRNA vaccines against SARS-CoV2 have been rarely reported, as the vaccines are overall very safe [28, 29]. For the first time in this case report landscape, we performed a statistical analysis on a cumulative sample.

The predictive value of anti-MDA-5 autoantibodies for the development of ILD/RP/ILD is not new. MDA-5-positive DM is a well-recognised subset of DM with known frequent severe pulmonary dynamics and high lethality [30–34]. The case report landscape can confirm this clinical phenotype of MDA-5-positive DM even after mRNA vaccination. In the context of the pathogenesis of MDA5-positive DM, a viral trigger has often been discussed [35]. Our results show that SARS-CoV 2 mRNA vaccines should also be discussed as potential triggers in the sense of molecular mimicry like SARS-CoV2.

In addition to the association between dermatomyositis and mRNA vaccination discussed here, there has been speculation about the induction of autoimmunity by mRNA vaccination in other organ systems. In addition to other myositides [36], including autoinflammatory myocarditis/pericarditis [37–42], reports of autoimmunity in the endocrine [43, 44], neurological haematological [45, 46] and cutaneous [47, 48] systems have been documented. Larger sample sizes are required for more precise statistical analysis.

### Limitations

The results of this work show only a correlative association, with no evidence of a causal pathomechanism. The association of the case reports with the vaccine can, in principle, also be attributed entirely artificially. The sample size does not allow parametric testing in many cases. A larger cohort is needed to better control for possible mediator variables. A certain reporting bias of the cumulative case reports must also be assumed. However, the general characterisation of the sample shows a certain comparability and representativeness. The definition of ILD/RP-ILD is not clearly differentiated in many case reports, so that we were also unable to make a more precise distinction for the case report landscape. In terms of laboratory chemistry, very different assays are used to detect autoantibodies, which also makes it difficult to compare cases. The fact that a malignancy was detected in part of the case report landscape suggests that paraneoplastic DM should still be considered as the most important pathomechanism, despite the suspected association with mRNA vaccination. In addition to the parameters of the case report landscape examined here, this study was unable to provide any insight into the suspected connection between IFN-γ signalling and autoantibody production against MDA5 and a SARS-CoV-2 infection. This was due to the inability to select suitable parameters from the case report sample to investigate this question. Further experimental or clinical work is required to measure IFN-γ signalling in a suitable manner, which will enable the investigation of this suspected connection.

### Conclusions and outlook

Anti-MDA-5 autoantibodies could serve as discriminative biomarkers between statistically more and fewer vaccine-associated cases of DM in the case report landscape of DM following SARS-CoV 2 mRNA vaccination. In addition to the predictive value for ILD/RP-ILD development, this antibody detection suggests an interferon type 1-dependent pathomechanism for vaccine-associated DM, analogous to SARS-CoV2 infection with pulmonary involvement. The group of MDA-5 negative DM in the case report landscape presents with greater heterogeneity overall and often shows a malignant association despite presumed induction by mRNA vaccines.

The results presented here on DM after SARS-CoV 2 mRNA vaccination serve as a model for further research in the field of oncological mRNA vaccination. A particular challenge will be to discriminate between paraneoplastic DM as an autoimmune symptom of oncological disease and possible mRNA-mediated DM. With sufficient sample size, it would be interesting to statistically investigate similar case report landscapes for the association of mRNA vaccination with other autoimmune diseases. For a more detailed understanding of mRNA vaccines, elucidation of the putative pathomechanism via an interferon type 1 immune response is essential. In a clinical context, it would be prudent to increase the sample size and verify whether the results reported here for the MDA5-positive DM subgroup can be replicated in an independent clinical cohort with a comparable effect size.

## Data Availability

All data supporting the findings of this study are available within the paper. The raw data supporting the conclusions of this article will be made available by the authors, without undue reservation.

## Abbreviations

ANA: Antinuclear antibodies
CCP: Cyclic citrullinated peptides
DM: Dermatomyositis
ENA: Extractable nuclear antigen
ILD: Interstitial lung disease
IQR: Interquartile range
IRF3: Interferon regulatory factor 3
jDM: Juvenile dermatomyositis
Jo-1: Histidyl–tRNA synthetase
LGP2: Probable ATP-dependent RNA helicase DHX58
MDA5: Melanoma differentiation-associated protein 5
Mi-2/NuRD: Nucleosome Remodeling Deacetylase complex
MVAS: Mitochondrial antiviral-signaling protein
NFκB: Nuclear factor ‘kappa-light-chain-enhancer’ of activated B-cells
NXP2: Nuclear Matrix Protein-2
OR: Odds ratio
PL-12: Alanyl-tRNA synthetase
PL-7: Threonyl-tRNA synthetase
PM/Scl100: Polymyositis/Scleroderma (100 kDa) antigen
PM/Scl75: Polymyositis/Scleroderma (75 kDa) antigen
RF: Rheumatoid factor
RNP: Ribonucleoprotein
RP-ILD: Rapidly progressive interstitial lung disease
SAE: SUMO-1 activating enzyme
Scl-70: Topoisomerase I
Sm: Smith antigen
SRP: Signal recognition particle
SSA(Ro): Soluble Substance A-antigen (Robert-antigen)
SSB (La): Anti-Soluble Substance B-antigen (Lane-antigen)
SUMO-1: Small ubiquitin-like modifier 1
TIF-1γ: Transcriptional intermediary factor-1γ
TRIF: TIR-domain-containing adapter-inducing interferon-β
